# Three Years with the Army of the Potomac—A Personal Military History

**Published:** 1911-11

**Authors:** William Warren Potter

**Affiliations:** Buffalo, N. Y., Brevet Lieutenant Colonel, U. S. Volunteers ; Surgeon in Charge of First Division Field Hospital Second Army Corps; Surgeon 57th Regiment New York Volunteers; Assistant Surgeon 49th Regiment New York Volunteers; Recorder Second Division Hospital Sixth Army Corps, etc., etc.


### Three Years with the Army of the Potomac—A Personal Military History

By William Warren Potter, M.D., Buffalo, N. Y., Brevet Lieutenant Colonel, U. S. Volunteers; Surgeon in Charge of First Division Field Hospital Second Army Corps; Surgeon 57th Regiment New York Volunteers; Assistant Surgeon 49th Regiment New York Volunteers; Recorder Second Division Hospital Sixth Army Corps, etc., etc.

### HARRISON'S LANDING.

We steamed out into the river after we were loaded up and cast anchor for the night, as our flag would not protect us from the shore batteries of the enemy after dark. We got up steam at daylight, and in a few hours hove to off Harrison's Landing. Soon after we came to anchor General McClellan boarded the Louisiana by steam launch, and proceeded to the cabin where two regular army officers, Major H. B. Clitz and Captain Chambliss, were lying upon cots. McClellan spent nearly an hour in low conversation with them, evidently for the purpose of ascertaining all he could of affairs in Richmond. General J. E. B. Stuart came almost daily to see these officers in Libby, sitting between their cots which were contiguous, and spending a short time in conversation with them. These two officers were wounded and captured at Gaines's Mills June 27th. Finally, the medical officer and nurses were put ashore and sent to their respective regiments, while the Louisiana proceeded north to distribute the wounded to the various hospitals.

I reached the camp of the 49th about two o'clock P. M. Saturday, July 19th, and everybody greeted me with kind words, anxiously inquiring after any of their friends in Richmond, and as to my treatment by the enemy. I hardly had time to get some dinner before General Davidson sent for me, and I spent an hour or more with him in telling him my experience, and in describing what I saw in Rebeldom. General Davidson was very gracious and seemed much pleased with the interview. He mentioned me in his report, besides giving me a letter to Governor Morgan recommending me for promotion.

July 20. I visited Dr. Hamilton at General Keyes's Headquarters, where he was serving as Medical Director, and gave him the first news he had received of Theodore. We spent an hour or more in agreeable conversation, and he thanked me very kindly for coming to see him with such good news. On my return from Richmond I found the regiment quite sickly—more so than it had been at any time before. Colonel Bidwell had gone home on sick leave a few days before my arrival, and Dr. Hall had applied for leave on account of sickness, which came back approved on the 20th. The paymaster arrived on the 19th to pay the brigade, and we were paid on the 22d, up to July 1. I sent home $150.00 by Dr. Hall on the 23d.

I left McLean in good spirits in Richmond, taking his imprisonment quite philosophically. He was wounded by three sabre cuts, two upon his head, laying open his scalp quite extensively, and another in one arm. They all healed kindly, after which he was put on duty in the office of the Commandant of the prison. About 200 officers were confined in the building with him, among whom were Generals McCall and Reynolds. Our camp at Harrison's was pleasantly situated on a knoll, about two miles from the Landing at Berkley, where we had a Regimental Hospital once more, the first since we came on the Peninsula. A cow—tell it not in Gath—was obtained for the use of the hospital, and thus we were afforded nice milk twice a day, which, together with fresh vegetables, made us seem like living again.

July 29. I heard from home today—the first letter in four weeks. Dr. Hall has promised to use his influence to secure a promotion for me while he is away, and it is rumored that Colonel Folsom is expected to command a regiment to be raised in Wyoming County, which, if true, may afford a chance of success.

July 30. The weather is very hot, and has so continued for several days, with no prospect of abatement at present. On the night of the 31st, the rebels opened their batteries from the south side of the river, attempting to disable our shipping at Westover, the lower landing. The gunboats soon replied, and after an hour or so silenced the enemy. The scene of the bursting of those immense shells in the middle of the night was grand, but no great damage was done to us.

August 3. We had rain which left the air cooler, and the health of the army begin to improve. Our regiment shows a smaller sick list by one-half than it did two weeks ago. This morning I report sixteen sick in hospital and twenty-seven in quarters, mostly cases of diarrhoea which is quite obstinate in character.

The attitude of the North in relation to the demand for more troops surprises us here in the army. There is very little use in teasing, or hiring, or buying soldiers to defend our country. Men must be had, and if they won't come voluntarily, they must be made to come. This Army, and Pope's, must be reinforced largely and speedily. Drafting must inevitably be resorted to sooner or later, and it might as well be done now as ever. It will never do to handle the rebellion with kid gloves, as there is very little Union sentiment remaining in the South, and we need have fear of injuring this supposed class by an aggressive warfare of the most unrelenting kind. These are some of the views entertained in the army at this time—views which are freely expressed on all hands.

August 6. The weather is hot again and our division is under orders to be ready to move at a moment's notice. The health of the regiment is very good at present and the sick list is daily diminishing. An Assistant Surgeon has been temporarily assigned to help me while Dr. Hall is away, which affords me some relief from the pressure of the routine duties. Captain Haines and Lieutenant Bull went home on sick leave today and Quartermaster Boyd went to Fort Monroe on business for the regiment.

August 9. I have been suffering considerably of late with pain in my front teeth, so much so, indeed, that my face is swollen quite badly. I have applied for leave to go to Baltimore and have them cared for by a dentist.

August 11. My face is better and my application for leave came back disapproved, which didn't surprise me much. (This paper is among my war files, and contains some interesting autographs). Everything is packed up in camp ready to move, and we are sitting around upon the boxes waiting for the orders to start. The name of my assistant is Dr. Meekley, of Pennsylvania. General Davidson has been assigned to another command and will not return to us; he goes to St. Louis, I believe. Captain Heacock has gone home on recruiting service.

August 12. It is very hot and we are still waiting to move, expecting the order momentarily. Preparations are going on all around us, which indicate a movement of the whole army. We shall see.

August 13. We are still hourly expecting, living out of doors, as our tents and camp equipage are all packed and the wagons have moved off. This suspense is more trying than the labors of an active campaign. Captain Hamilton of the 33d returned from Richmond today and reports McLean, who was released with him, as off for Fort Monroe and probably the North. The weather is cooler, but I feel very much depressed in spirits—don't know why.

August 14. We are not off yet—waiting, waiting, waiting. McLean, much to our surprise, came to our camp (?) yesterday, and is now staying with his brother-in-law, Tom Dewey. General Smith has just returned from twenty days' leave spent in the North, and comes back a Major General, which we all think he deserves as much as any man. He has taken his division through some severe campaigning, and always skillfully, with little loss and no disaster. He is cool under fire, and altogether an ideal division commander.

ALEXANDER—POPE.

August 21. After six days hard marching we are at Hampton preparatory to embarkation, though we are unadvised as to our destination. The march down the Peninsula, especially that part of it between the camp we left at Harrison's and the Chickahominy, was so hot and dusty that we all looked like millers just out of duty in a flouring mill. The flies, too, perplexed us greatly the first day; so much so, indeed, that we were obliged to carry branches cut from trees to brush them off our horses, as well as ourselves. We passed over the old battle-field of Williamsburg and through the heavy works at Yorktown, places of familiar and historic interest. We embarked at Fort Monroe, Friday, August 22d, on the steamer C. Vanderbilt, and reached Alexandria Saturday evening, remaining on board all night and disembarking this morning (Sunday, August 24). The regiment was sent to camp about two miles out of Alexandria on the Fairfax Road, and I obtained a pass and took the boat for Washington. O. H. Dorrance, of Attica, a military telegraph operator, went with me, and we staid all night at the National Hotel. I had known Dorrance in my youth; he was a good companion, and we had a pleasant visit discussing the times of our boyhood days.

August 25. I returned to camp this morning and find that our horses have not yet arrived from the Peninsula. It looks as though we would be sent out to assist Pope who, I fear, is getting into some difficulty. On the boat coming from Fort Monroe to Alexandria I lost my baggage—a satchel containing my clothing and some valuable papers, including my rebel passes, etc.; so I busied myself Tuesday, the 26th, in replenishing my scanty wardrobe at Alexandria, buying, however, only such articles as I stood in immediate need of. Dr. Hall and Colonel Alberger returned from sick leave the 26th, both looking well, and Major Johnson has now forwarded his application for leave. We are located by the roadside between Forts Ellsworth and Lyon, and it is so dusty that we suffer considerably therefrom. As we are without horses we are obliged to stay in camp or go about on foot.

Friday, August 29. Franklin's corps, consisting of Smith's and Slocum's Divisions, was ordered to go to the scene of Pope's operations supposed to be somewhere near Manassas, and whose guns we had been hearing for a day or two. We were obliged to start without our horses, which was a serious inconvenience; but Dr. Hall and I kept up with the troops the first day, as the march was not a long one. We slept together that night under an apple tree in an orchard near our bivouac, without covering, save a borrowed blanket. Saturday morning we moved on early, but Dr. Hall and I were obliged to lag behind for a time. However, we soon found an ambulance going to the front, the driver of which was persuaded to give us a ride, thus enabling us to overtake the regiment Saturday evening between Centerville and the second Bull Run battle field, the closing scenes of which engagement were just then being enacted.

August 31. Sunday morning found us all back at Centerville, Pope and the Army of the Potomac as well, in the midst of a drenching rain, without any place to even sit down upon excepting the mud. We remained there until Monday night, when we fell back to Fairfax C. H. Monday P. M. A severe thunder storm set in, and I could find no shelter save the canvas covers of the artillery, and this was not safe on account of the danger from lightning. That night, after we reached Fairfax, I broke open a store and lay down upon the counter at three o'clock A. M., sleeping until daylight. Tuesday, September 2, we remained near Fairfax, marching and counter-marching all day and at night returning to our former position near Fort Lyon, from which we set out five days before to join Pope. Our horses met us Wednesday morning just as we were approaching Alexandria again, so we had no use of them during this fatiguing but fruitless affair. To add to my discomfort I had on a pair of new boots when I left Alexandria and was, as a matter of course, tortured by them more or less during the whole trip.

### THE MARYLAND CAMPAIGN—ANTIETAM.

September 4. Today McClellan is assigned to the defense of Washington, which brings us once more under his command. How long it will last or what next I cannot say; but, God be praised for delivering us from Pope. Dr. Jenkins reported to us as 2d Assistant Surgeon on the 5th. He had formerly been in the employ of Dr. Potter, of Cowlesville, and brought news of the people in that region.

September 6. Saturday, P. M., we crossed the Potomac over Long Bridge, marched through Washington and Georgetown out the Tennallytown Road, and bivouacked for the night near the latter village. This was the beginning of the Maryland Campaign, for news had now been circulated that the rebels had crossed into that state above Harper's Ferry. It was late in the evening when we passed through Washington, but the people were up to greet us and we were received with demonstrations of enthusiastic applause at almost every corner, especially at Willard's Hotel, where a large crowd had collected. The people evidently regarded the Army of the Potomac as their salvator and were grateful that it stood—a mighty barrier —between them and the rebel horde. As we passed General McClellan's residence we gave nine rousing cheers, which were taken up by each regiment as it passed. At a number of places during our passage through the city, I observed ladies supplying the soldiers with water, always so needful upon the march, and particularly in hot weather.

September 7. We lay in bivouac above Tenallytown until late Sunday P. M., and then set out towards Rockville, Md. Soon after starting we came to a stream, a small creek which I think was Rock Creek, where stood a carriage near the ford, in which sat President Lincoln, Mr. Seward, the Secretary of State, and Mr. Stanton, the Secretary of War. The soldiers cheered them lustily, and they all acknowledged the attention with bared heads and waving hats. We marched on by moonlight until eleven o'clock P. M., when we again went into bivuouac.

September 8. Monday was hot and dusty, and, though we did not start very early, we kept it up until late at night to make up. This was a good plan, for it enabled us to avoid the great heat of midday; and so through the week about after this fashion. On the 11th we bivouacked at Barnesville, and by Saturday, the 13th, we had reached Jefferson, where we again bivouacked for the night.

September 14. Sunday morning the sun rose bright, betokening a hot day, and after a hasty breakfast we started on the road to Crampton's Gap in the South Mountain, that was visible in the distance. We soon began to hear firing and see the smoke of battle at Turner's Gap, a few miles to our right; but the Sixth Corps was destined to engage in a battle of its own at Crampton's, later in the day. About noon we rested in a piece of woods and cooked coffee; Slocum's division being in advance, pushed on up to the Gap, we of Smith's soon following. Just at night we reached the foot of the mountain, when we were welcomed by a few shots from a battery belonging to Howell Cobb's brigade, stationed at its summit. The guns could not, fortunately, be depressed enough to get our range, so most of the projectiles passed over us without damage. It was quite dark by the time we were fairly ascending the mountain, and, hearing the groans of wounded men in the woods off the road, Dr. Hall and I dismounted and went to their relief. We found a number of rebels scattered through the woods, most of whom were wounded in the abdomen or chest, mortally. After ministering to them the best we could, we sought the regiment, which had passed on over the mountain, and was in bivouac on its western slope. We reached it late at night, tired and worn by the fatigues of the day, and slept soundly on the ground.

September 15. Monday morning the Sixth Corps was ordered to go to the succor of Harper's Ferry, but had not gone far before an order came, calling us back, that Post having already surrendered. We remained in camp near Rohrersville during the balance of Monday and all day and night of Tuesday. Tuesday, the 16th, was the anniversary of our departure from Buffalo; what memories clustered around the day; what a year of camping, marching, and fighting we had seen—and the end was not yet. Dr. Hall and I visited the South Mountain battle field at Turner's Gap, on this Tuesday P. M. It was a beautiful day, and the scenery was something indescribably grand. Before us, looking east, was the Frederick Valley, with the city nestling in its midst a few miles away; behind us lay the Washington Valley, equally picturesque, but where even now firing was going on between some of the contending forces, manoeuvring for position near the Sharpsburg field. Some of the enemy's yet unburied dead lay at our feet as we were sweeping the horizon with our and war. We reached our regiment in the early evening with glasses, and altogether it was a sadly beautiful picture of peace appetities for supper sharpened by its keen air, which we soon partook of and then lay down to sleep and rest, preparatory to the coming of the morrow with all its dreadful carnage.

September 17. Wednesday morning we awoke early and prepared to march, in the midst of a misty rain. I shaved myself while my tent was being struck, which is the last time my upper lip has felt the touch of a razor. We were off betimes, and soon passed through Rohrersville en route to the Antietam field, which we reached about noon. The Sixth Corps was thrown in on the right to relieve Sumner, and the Third Brigade of Smith's Division, commanded by Colonel Irwin, 49th Pennsylvania Volunteers, made a charge on the enemy at the Dunker Church on the Hagerstown Road. (Illustrated in the Century, June, 1886, Page 298). Dr. W. J. H. White, Medical Director 6th Corps, was killed by a sharpshooter while sitting on his horse very near General Franklin—the first casualty in the Corps in this fight. The 49th lost two killed and twenty-one wounded; among the latter Lieutenant Colonel Alberger, struck in the face by a fragment of a shell. He was taken to the 2d Corps Hospital, as the Sixth Corps did not establish separate hospitals for itself, and I spent part of the day with him there on the 18th. During this day there was no fighting to speak of and everybody who could do so rested as much as possible.

September 19. The enemy retreated during the night of the 18th, and on Friday morning we found he had escaped to the other side of the river, where he stood at bay. I visited the famous "Sunken Road," where I saw the dead lying in swaths, and in the corn-field they were also thickly strewn; the number of dead bodies that I saw must, altogether, have aggregated near five hundred. I counted eleven dead near a caisson that fell into our hands. During the next few days we moved about considerably, but never very far at one time. On Sunday, the 21st, we marched to the vicinity of Williamsport, where our baggage came, so that night I enjoyed the luxury of a tent once more. On the 23d we moved about four miles down the Potomac and camped at Dam No. 4, to guard it, which is about half way between Williamsport and Sharpsburg. On the 22d I rode twelve miles to General McClellan's headquarters, to obtain Colonel Alberger's leave, and I brought it back with me duly approved; so he went home rejoicing that his wound was no worse. These were bright days for us, for we felt the influence of the victory gained at Antietam; the weather, too, was pleasant, though the nights were cool. The old troops are very much worn in vigor, besides being poorly clad, and are particularly suffering for shoes. The country we are in is the finest we have seen in the South, resembling New York more in its thrift and enterprise; and under its genial sun the health and stamina of the army is improving. On the 27th, being still at Dam No. 4, we were reminded that it is just a year since we first crossed the Potomac into Virginia.

September 29. We are still at Dam No. 4, which is a longer time than we have been in any one place since we left Harrison's Landing. I am very short of money, having had no pay since July, and no prospect of getting any at present, as the government coffers are reported as nearly empty. Rumors are afloat that we go to Hagerstown soon, which I hope are true, for that is said to be a delightful region. Our mails are very irregular, sometimes a week apart, for which there seems no good reason. We are certainly in a more accessible region than Virginia, but we do not fare as well in regard to our mail facilities as we did there.

October 1. Today I attended the funeral of one of our men, the first that has died in camp since we left Camp Griffin. Others have died, but at the different hospitals where they have been sent. We were surprised by the receipt of a mail today, with letters from home to the 19th ult. Rain fell today, for the first time in many weeks, which was refreshing in every sense of the word.

October 3. Friday, we were reviewed by the President and General McClellan, which is the first review we have had since we were at Harrison's Landing. The Sixth Corps did itself credit as usual and the affair passed off pleasantly. We have a new Brigade commander in place of General Davidson, who, it will be remembered, left us at Harrison's Landing. Brigadier General Francis L. Vinton, nephew of the Rev. Dr. Vinton of New York, is our new General. He has just been promoted from Colonel of the 43d New York Volunteers, is a young looking man, but may do well.

October 8. We received fifty-four recruits from Buffalo today, which is a substantial and much needed addition to our wasted ranks. General McClellan's headquarters were moved to Harper's Ferry today, which is thought by some to indicate a forward movement soon. Major Johnson has been in command of the regiment since Colonel Alberger's wound; he is a cool, brave officer, but not generally popular. I like him very much myself, for I know him well—probably better than any other officer, for he has no intimates. Tom Dewey was wounded in the heel at Antietam, but is on duty and is well.

October 10. We have just been surprised again with a mail, the first in ten days, which is a welcome change. Letters and papers from home are our great consolation, and when we are deprived of them so long it seems as if life were a blank. Mr. Woodruff, the sutler, has just arrived in camp, the first time he has been with us since Alexandria. We still remain quiet, having occupied this camp at Dam No. 4 about seventeen days. We are midway between Sharpsburg and Williamsport, and nine miles from Hagerstown, in the very garden of Maryland. Generals Franklin, Smith and Brooks have their wives with them at Hagerstown, which is a very pretty place according to report.

October 11. About one o'clock this morning Major Johnson came to my tent, saying we were ordered to move immediately. It was raining at the time and there was much mystery in the suddenness of the movement in the middle of the night, when we had not seen a rebel for nearly three weeks. However, we started at four o'clock A. M., and at nine passed through Hagerstown. We then learned that Stuart had made a raid around our rear, burnt stores at Chambersburg, and that we were being sent out to aid in intercepting him. But, he was too nimble and fleet-footed for infantry, even eluding our cavalry and passing over the Potomac at Point of Rocks without interruption, after making the complete circuit of the Army of the Potomac for the second time in four months. Our services, therefore, being of no further use, we went into camp in a beautiful woods one mile and a half from the town, and on the 13th Dr. Hall and I visited the city for the first time. The nights were beginning to grow cool, and we, therefore, availed ourselves of this opportunity to purchase a small cast iron stove, which we brought home with us and set it up in our tent that night, having the first inside fire of the season. The popularity of the scheme was attested by our numerous visitors, not only this evening, but on various other occasions during the next few weeks.

The 3d Brigade has a brass band again, which is the only one in the division at present. The bands were all mustered out on the 1st of August and we have had no music since until now, excepting such as the fife and drum furnished. Tom Dewey has not been well for a day or two, but seems better this evening and sits by my fire enjoying its benefits and comforts.

October 15. Dr. Jenkins has been quite ill for the past week, and I imagine he is not over charmed with military life. A little sickness is apt to remove all illusions as to the delights of camp, especially in an active campaign. Dr. Mulford has just returned from an absence of thirty days, having obtained his leave through the influence of Judge Levi C. Turner, of the War Department. He calls two or three times a day and is feeling quite happy.

October 18. The weather is beautiful—like Indian Summer—and today Dr. Mulford and I went into Hagerstown, where we saw the ladies promenading the streets, the shops busy, and all looked like civil life again. We also saw Mrs. General Franklin and Mrs. General Smith at the Sixth Corps Headquarters, where everything was gaily festive. General Slocum has been assigned to the command of General Banks's corps and General Couch has been ordered to Hancock, Md., today. Colonel Bidwell has returned to camp, having reported yesterday, and is looking quite thin for him. Today I was assigned to the medical charge of the 77th N. Y. Volunteers, as it was without a medical officer, the Surgeon and Assistant Surgeon both being away on sick leave. Tom Dewey has been ordered home on recruiting service, to relieve Captain Heacock, who has been absent sometime on that duty.

October 22. We moved in the night to Clear Spring, several miles towards Hancock, where we remained a week, guarding one of the dams or fords. While at Clear Spring about twenty officers boarded at the hotel there, which we enjoyed very much, as it was a pleasant change. We were all without money, but the landlord kindly trusted us until we should get our pay, the Quartermaster becoming responsible. I had charge of the 77th the week we were there. On the 29th we broke camp at three o'clock P. M., and moved to Williamsport, where we arrived at eleven o'clock at night, and remained during the 30th. Colonel Bidwell is with the 49th, but not on duty yet, as he is not feeling strong enough to assume command.

October 31. We moved from Williamsport to Boonsborough today, about twelve miles, where we arrived at noon, and then had our bi-monthly inspection and muster for pay. Mrs. General Pratt accompanied her husband on the march today and tonight they are at the U. S. Hotel in Boonsborough. Our brigade band is serenading them this evening, the weather being warm and the night delightful. General Pratt was formerly Colonel of the 31st New York, the regiment that Dr. Hamilton went out with as Surgeon. (General Pratt is now judge of one of the courts in Brooklyn, where he resides). We are ordered to move at six o'clock A. M. tomorrow, but whither bound we do not know—some say to Harper's Ferry, others to Frederick—but the morrow will tell.

November 2. We made a rapid and long march yesterday from Boonsborough to Petersville, passing through Turner's

Gap where Reno and Burnside had their battle on September 14, and then through Birkitsville, at the foot of Crampton's Gap, where Franklin's Sixth Corps was engaged the same day; but all evidences of the carnage had disappeared, save only the bullet marks on the trees and fences. We are two miles and a half from the Potomac, and six miles below Harper's Ferry. A hospital was established for the Sixth Corps at Hagerstown and we left 700 sick there. We expect to cross into Virginia tomorrow and then farewell to the delightful scenes of southwestern Maryland—My Maryland—where we have had so much of joy and sadness commingled. We have now been campaigning for eight months, which has worn us down in body and discouraged us in mind to a considerable degree, and I think we all dread going back into Virginia, where the old ground is evidently to be fought over again.

(Continued.)

